# Promiscuity of lanthipeptide enzymes: new challenges and applications

**DOI:** 10.1007/s11274-025-04505-5

**Published:** 2025-08-06

**Authors:** Carlos García-Ausencio, Fernando Guzmán-Chávez, Romina Rodríguez-Sanoja, Sergio Sánchez

**Affiliations:** 1 Instituto de Investigaciones Biomédicas, Universidad Nacional Autónoma de México (UNAM), México City, 04510 México; 2 Faculty de Chemistry, Food Science and Biotechnology, Universidad Nacional Autónoma de México (UNAM), México City, 04510 México

**Keywords:** Lanthipeptides, Enzymatic promiscuity, Leader peptide, Posttranslational modifications

## Abstract

Lanthipeptides are a group of peptides synthesized by ribosomes that undergo post-translational modifications and have significant potential for medical and biotechnological applications. Various bacterial strains produce these peptides, and their synthesis involves the structural modification of precursor compounds through specialized enzymes present within a biosynthetic gene cluster (BGC) of the producing organisms. These enzymes are particularly notable for their capacity to modify non-cognate substrates, allowing for the installation of lanthionine rings on precursor peptides and enabling further modifications, such as methylation, reduction, and oxidation, to enhance the biological properties of specific peptides. The inherent flexibility of lanthipeptide enzymes—an important characteristic of this class of proteins—can be utilized to create peptides with improved bioactive and physicochemical properties. This review synthesizes recent advances in the application of promiscuous enzymes for the synthesis of bioactive peptides, emphasizing the diverse classes identified to date.

**Graphical abstract:**

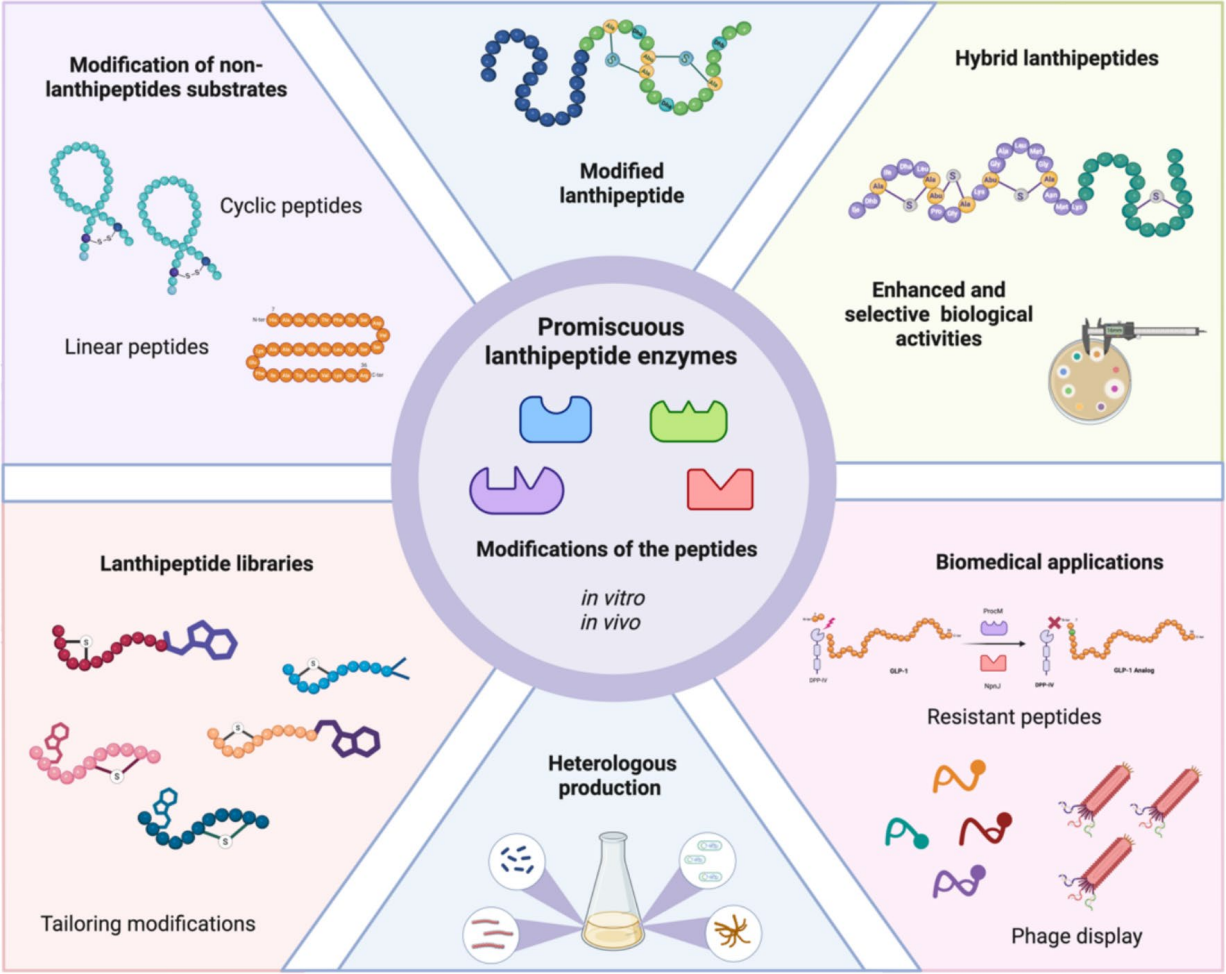

## Introduction

**Fig. 1:**
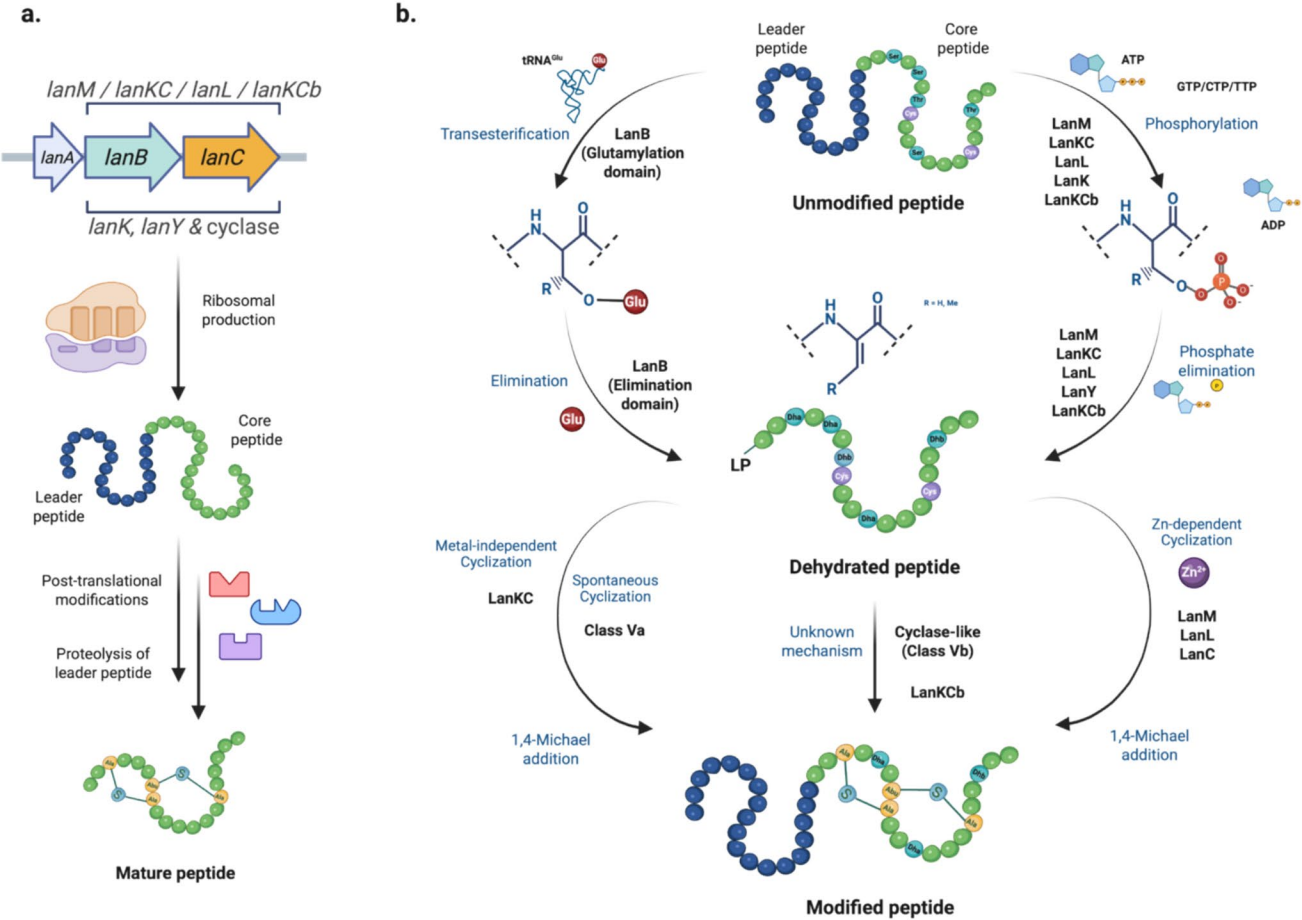
The processing of lanthipeptides: **a** The biosynthetic pathway of lanthipeptides; **b** the general mechanisms of lanthionine ring formation across all known lanthipeptide classes today

Gram-positive bacteria mainly produce lanthipeptides, but bioinformatic analyses reveal many biosynthetic gene clusters for lanthipeptides in Gram-negative bacteria and archaea. This is notable given the limited number of characterized lanthipeptides in these groups (van der Donk and Nair 2014; Barbosa et al. 2015; Caetano et al. 2020; Walker et al. 2020; Castro et al. 2021; Ramírez-Rendón et al. 2023; Roblero-Mejía et al. 2024). These peptides are noted for their biological properties, particularly their antimicrobial activity against multidrug-resistant bacteria (MDR), such as Methicillin-resistant *Staphylococcus aureus*, vancomycin-resistant enterococci (VRE), and various clostridia strains (Singh et al. 2020; van Staden et al. 2021; Shi et al. 2022; Deisinger et al. 2023; Wang et al. 2025). In addition to their well-documented antimicrobial effects, lanthipeptides have also been found to possess antiviral (Blockus et al. 2020; Fu et al. 2021; Singh et al. 2023), antifungal (Mohr et al. 2015; Singh et al. 2023), and other significant biological activities (Iorio et al. 2014; Han et al. 2022; Wang et al. 2023). Several studies have explored even their potential as diagnostic molecules due to their ability to bind to cell membrane components, such as phosphatidyl ethanolamine in eukaryotic cells (Luo et al. 2016). Nisin (NIS) is a lanthipeptide that has been widely used as a food preservative for over fifty years due to its strong activity against food pathogens, including *Listeria monocytogenes* (Gharsallaoui et al. 2016; Ibarra-Sánchez et al. 2020). This knowledge positions lanthipeptides as promising candidates for various clinical and biotechnological applications (Ramírez-Rendón et al. 2023). These peptides are categorized into the ribosomally synthesized and post-translationally modified peptides (RiPPs) and are distinguished by the presence of lanthionine (Lan) and methyl-lanthionine (MeLan) rings in its structures (Repka et al. 2017; Hudson and Mitchell 2018). The precursor peptide (PP) is divided into two regions: the leader peptide and the core region, and it is encoded by a biosynthetic gene cluster (BGC) (van der Donk and Nair 2014; Repka et al. 2017). The biosynthesis of lanthipeptides starts with the production of the peptide by ribosomes. This is followed by the binding of the leader peptide (LP) to specific biosynthetic enzymes. These enzymes then carry out post-translational modifications on the core peptide (CP). Finally, a protease cleaves the LP, resulting in a mature peptide that is responsible for the compound's biological activity (Fig. 1a). Currently, six distinct classes of lanthipeptides have been identified, each characterized by specific enzymes that facilitate dehydration and cyclization reactions to form Lan and MeLan rings (Xu et al. 2020; Lagedroste et al. 2020; He et al. 2023) (Fig. 1b). These enzymes display broad substrate specificity, often referred to as substrate tolerance or substrate promiscuity. This concept is a form of enzymatic promiscuity, where a single enzyme can catalyze the same chemical reaction on different substrates (Hult and Berglund 2007). The term was first used in the context of lanthipeptide biosynthesis over two decades ago, specifically in studies of the NIS biosynthetic cluster, which was employed to install rings in non-lanthipeptide substrates (Kluskens et al. 2005). This concept was later supported by other biosynthetic enzymes, such as lacticin 481 synthetase (Kuipers et al. 2004; Chatterjee et al. 2006; Zhang and van der Donk 2007; Rink et al. 2007). Since the discovery of prochlorosins (Li et al. 2010), a class II lanthipeptide composed of 29 precursor peptides (PPs), new evidence has emerged showing that biosynthetic enzymes can modify multiple PPs with varying structural features. These enzymes are referred to as promiscuous enzymes (Tang and van der Donk 2012), highlighting their ability to work with a relaxed range of substrates. Both terms are currently used to describe the ability of these enzymes to catalyze the same reaction (such as dehydration or cyclization) across different peptides, even if those peptides do not belong to the original cluster. This characteristic is often associated with the LP involved in the process of enzymatic recognition (Majchrzykiewicz et al. 2010; van Heel et al. 2016). Additionally, the presence of motifs and secondary structures is crucial for the correct processing of the products, although this role varies according to the lanthipeptide class (Plat et al. 2013; Hegemann and van der Donk 2018; Wiebach et al. 2020). A conformational model has been proposed, where the LP binds to the biosynthetic enzyme, thereby improving the affinity to CP due to the observed low affinity of these enzymes to CP in various systems. This feature allows the modification of different substrates (Levengood et al. 2007; Oman et al. 2012; Abts et al. 2013; Thibodeaux et al. 2015; Rahman et al. 2020). Therefore, understanding these characteristics is essential for various applications. From the modification of mutational cognate-peptides (Zhang and van der Donk 2007) to forming rings on medically important peptides such as angiotensin and erythropoietin (Kluskens et al. 2005) to improve their stability, the use of promiscuous enzymes allows the fast production of new and improved peptides. A study conducted in 2016 used NIS machinery to produce five new lanthipeptides with high antibacterial activity without the need to be modified by their cognate enzymes, reducing challenges by using already known systems (van Heel et al. 2016). Even, the broad substrate tolerance of ProcM has been used to produce lanthipeptide libraries. The search for inhibitors of HIV p6 protein in human cells, led to the creation of a lanthipeptide library of 10^6^ peptides using the same LP (Yang et al. 2018), supporting the great usefulness of promiscuous enzymes to produce new peptides of biotechnological importance. At the same time, we cannot ignore the usefulness of tailoring enzymes to diversify to lanthipeptides. The protease ElxP and the short-chain dehydrogenase ElxO, from epilacin 15X biosynthesis, demonstrated relaxed substrate specificity to cut NIS and reduce lactocin S (a class I lanthipeptide), respectively, supporting the fact that tailoring enzymes can also be promiscuous enzymes (Ortega et al. 2014).

In this review, we summarize the latest research advancements in the use of promiscuous enzymes to produce new natural products and their potential biotechnological applications, with a specific focus on lanthipeptides. We also highlight key properties of the biosynthetic enzymes involved in lanthipeptide production.

### LanB and LanC enzymes

Biosynthesis of class I lanthipeptides is catalyzed by two primary enzymes: a dehydratase, LanB, and a cyclase, LanC. In general, these enzymes are functional in several heterologous hosts like *Escherichia coli*, several actinobacteria, *Lactococcus* sp., *Bacillus,* and in vitro systems (Foulston and Bibb 2010; Sherwood et al. 2013; Liu et al. 2020; Zhao and Kuipers 2021; Chen et al. 2021; Chen and Kuipers 2022; Kaweewan et al. 2022; Lee et al. 2023). Most studies on the enzymatic mechanisms of this class of lanthipeptides have concentrated on the NIS enzymes, NisB and NisC. This system has advantages over other enzyme systems, such as those found in actinobacteria (for instance, the microbisporicin biosynthetic system), because it effectively catalyzes dehydration and cyclization reactions without the need for NisB or NisC to function in concert (Ortega et al. 2016; Repka et al. 2018). Importantly, both NisB and NisC require a LP for catalyzing ring formation. Research on NisB and NisC has highlighted the significance of the FxLx motif in the LP, as well as the STKD (−12 to −19) and PR (−2 to −1) motifs for binding to the precursor peptide (Plat et al. 2011; Mavaro et al. 2011; Khusainov et al. 2013; Repka et al. 2018). Interestingly, functionality with leaderless peptides has been demonstrated *in vivo* when an antimicrobial product was produced in *Lactococcus lactis* NZ9000. This reaction was enhanced when the LP was expressed in *trans*, resulting in fewer premature nisin molecules and increased antibacterial activity (Khusainov and Kuipers 2012). These findings highlight the crucial role of the LP in binding biosynthetic enzymes, particularly NisB, which exhibits a greater affinity for the LP compared to NisC. Additionally, in this system, specific mutations in the LP are more easily tolerated (Rink et al. 2005; Plat et al. 2011; Abts et al. 2013). This flexibility allows for the incorporation of identification and purification tags on the N-terminal side, even for larger peptides.

Recently, it was reported that fluorescence proteins such as mCherry red fluorescent protein are well tolerated by the nisin and clausin machinery, as well as the green fluorescent protein (GFP), which was added to LP of both NIS and Pep5 in *E. coli*, where the peptides were successfully processed and the mature peptides remained active (van Staden et al. 2019; Van Zyl et al. 2023).

**Fig. 2:**
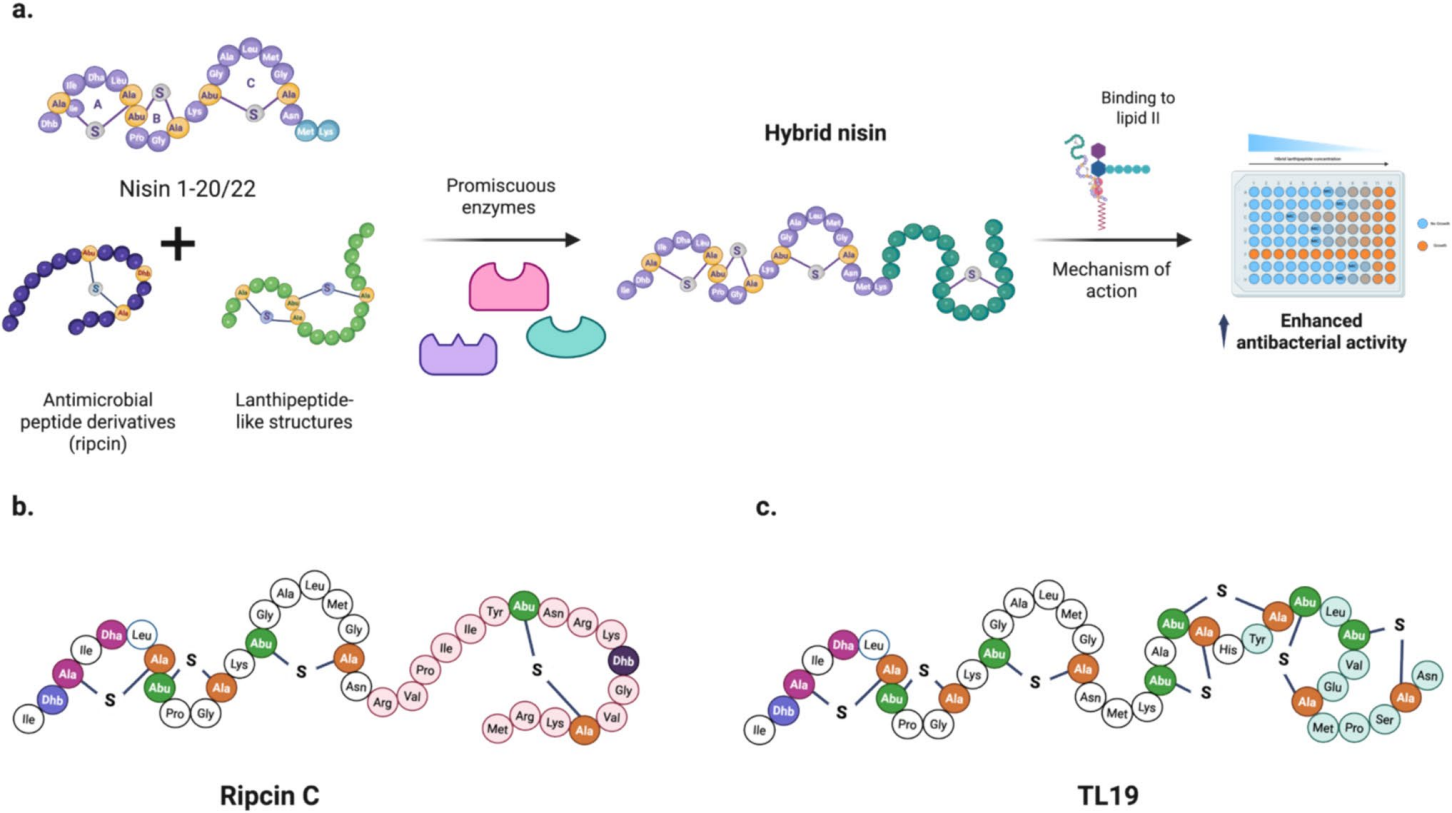
Production of hybrid NIS peptides for new peptide variants. **a** General overview of the production of hybrid peptides. The NIS 1–20/22 segment represents the lipid II-binding site, while a non-cognate peptide is joined at the C-terminal end. **b** The structure of Ripcin C is depicted as a hybrid peptide combining NIS 1–20 (shown in white) and ripcin (shown in pink), which is a derivative of an antimicrobial peptide (Zhao and Kuipers 2021). **c** The proposed structure of TL19 is presented as a powerful hybrid lanthipeptide that merges NIS 1–22 (in white) and HalA1 (in green), both of which correspond to lipid II binding sites (Zhao et al. 2020b)

NIS was the first lanthipeptide identified in lactic acid bacteria (LAB) (Delves-Broughton et al. 1996; Field et al. 2023). It is well-researched and documented. Due to its antibacterial properties against Gram-positive strains, it serves as a prototype for lanthipeptide engineering. Regarding its biosynthetic pathway, although the NisB and NisC enzymes are mainly involved in the production of NIS, other proteins, such as the ATP-binding cassette (ABC) transporter (NisT) and the subtilisin-like serine protease (NisP), are also related to the export and proteolysis of mature NIS (Cheigh and Pyun 2005; Lubelski et al. 2008). On the other hand, proteins related to resistance and immunity are also involved in the production of this lanthipeptide. The NisR and NisK are the key regulators in triggering NIS synthesis (Engelke et al. 1994; Qiao et al. 1996). In this sense, the NIS-controlled expression system (NICE) is utilized for several antimicrobial peptides, which use the NIS biosynthetic enzymes to induce lanthipeptides. The first characterized strain for NIS production was *Lactococcus lactis* subs. *cremoris* MG3163 (later re-named as NZ900). It was described that the insertion of *nisR* and *nisK* genes into the genome of the strain triggered the production of a cloned product in a plasmid with NisR promoter, using lower amounts of NIS (0.1–5 ng/mL) (Mierau et al. 2005; Mierau and Kleerebezem 2005). Likewise, other similar systems, controlled by subtilisin, are not limited to NIS utilization. Thus, it can be used by *Bacillus subtilis* (SURE) for the production of β-glucuronidase and green fluorescent protein (GFP) reporters, utilizing the promoter PspaS, and the regulatory genes *spaR* and *spaK* (Bongers et al. 2005). The NICE system was utilized to produce a wide variety of biomedical and industrial products, as well as specific class I lantibiotics (antibiotics containing lanthionine rings) to produce bioactive peptides (Mierau and Kleerebezem 2005; Montalbán-López et al. 2017; Sandiford 2020; Frelet-Barrand 2022). Over the years, these proteins have demonstrated great flexibility in accepting non-wild substrates, provided that the PL sequence in the peptides is preserved, with variations allowed at specific sites within the CP (Table 1). For instance, the antimicrobial peptides (AMPs) thanatin and rip-thanatin, effective against Gram-negative bacteria, are sulfur bond-containing compounds produced by the insects *Podisus maculiventris* and *Riptortus pedestris*, respectively. These peptides were used as peptide backbones to create a peptide incorporating a MeLan ring using the NICE system in *L. lactis* NZ900, forming thanacin and ripcin (Zhao and Kuipers 2021). Another example is the hybrid peptide known as RipC, which is created by combining the lipid II-binding region of NIS (comprising the first 20 amino acids) with ripcin (Fig. 2a). This peptide has demonstrated effectiveness against MRSA and *Acinetobacter baumannii,* displaying selective binding to lipid II (Zhao and Kuipers 2021) (see Fig. 2b).

**Table 1:** Recent reports on peptides produced by class I and II enzyme promiscuity. In general, their bioactivity and stability are improved

Class	Modifying enzyme	Peptide/Product	Characteristics	Bioactivity or relevance	Reference
I	NisBC	RipC	A hybrid peptide consisting of NIS (1–22) and ripcin is derived from rip-thanatin. The NIS (1–22) region binds to lipid II	Antibacterial	(Zhao and Kuipers 2021)
		Rombocin A	A short variant of nisin	Antibacterial and resistant to proteases	(Guo et al. 2024d)
		TL19	Hybrid peptide composed of the N-terminal sequence of NIS (1–22) and the C-terminal sequence of HalA1	Powerful antibacterial	(Zhao et al. 2020b)
	BalBC	Balucin-nis3	In Balucin, the fourth ring was changed to ring three of the NIS	Antibacterial	(Fu et al. 2023)
II	ProcM	15RGD	Modifications in the prochlorosin precursor, ProcA2.8, result in resistance to trypsin proteolysis	Affinity to αvβ3 integrin	(Hegemann et al. 2019)
		GLP-1 analogue	A glucagon-type peptide that is subsequently reduced by the dehydrogenase NpnJ	Resistant to DPP-IV	(Larsen et al. 2024)
	SyncM	SA2.3 and SA7.2	Positively charged peptide derivatives are linked to SyncA	Antibacterial	(Arias-Orozco et al. 2023)
		LACM and LACPINM	Derivatives of peptides related to lactoferrin		
				Antibacterial	
	CinM	Lantha analogue	An antimicrobial peptide analogue containing disulfide bonds is later modified with lipids		(Zhao et al. 2021)
				Antibacterial	
	MutMT	Nukacin Spp.2	An analogue peptide like nukacin is produced in *Streptococcus mutans* T8		(Biswas 2023)

Variations of the NICE system have been reported in recent studies. For example, Guo et al. (2024d) developed an NIS analog that is 25 amino acids shorter, derived from *Romboutsia sedimentorum*, and named it rombocin A. By utilizing the main enzymes involved in the NIS biosynthesis (NisBCT) from *L. lactis*, they modified rombocin A to create rombocin K. This modified peptide demonstrated increased resistance to proteases compared to NIS and exhibited enhanced bactericidal activity against *Listeria monocytogenes*. Similarly, Zhao et al. (2020a, b) utilized the NIS biosynthesis system to synthesize a novel lantibiotic with two lipid II binding motifs, designated as TL19. This hybrid peptide comprises the first 22 amino acids of NIS and a conserved domain, CTLTXEC, which is derived from two lanthipeptides: haloduracin (HalA1) and lacticin 3147 (LtnA1).

TL19 proved to be 64 times more effective against strains of *Enterococcus faecium*, including vancomycin-resistant enterococci (VRE) and ampicillin-gentamicin-resistant enterococci (AGRE), with a minimum inhibitory concentration (MIC) that was 64-fold lower than that of NIS. It also showed significant activity against *Acinetobacter baumannii* (Zhao et al. 2020b). The use of NIS fragments presents an attractive strategy for designing peptides with improved characteristics. Cerocin V is a new peptide developed from the combination of three mutated NIS rings (1–19) and one ring from rombocin. By eliminating the hinge region of NIS and mutating Arg to Val in the C ring of NIS, cerocin V demonstrates greater resistance to trypsin and chymotrypsin, as well as increased stability in plasma (Guo et al. 2024c), demonstrating the utility of this technique.

It is widely known that the main biosynthetic enzymes of NIS, NisB, NisC, and NisT, act cooperatively for NIS production; however, it is also possible to apply them to another biosynthetic complex. Chen and Kuipers (2022) studied the NisBC complex with the subtilin transporter SpaT from *B. subtilis*. When suppressed, the SpaBC complex (for subtilin production) was unable to produce NIS, probably because it did not recognize the LP of NIS. However, the transporter SpaT was able to transport NIS to the extracellular space. This research emphasizes the use of non-natives’ transport to export lanthipeptides, such as SpaT, which could have low substrate recognition. In the same study, the cyclase enzyme SpaC was able to cycle the NIS through LP exchange, confirming the importance of the LP (Chen and Kuipers 2022). However, the cyclase enzyme has a limitation regarding the presence of negatively charged amino acids near cysteine residues, which can reduce its productivity, as demonstrated in NisC. In 2023, a new lanthipeptide (named Balucin) from *B. subtilis* 168 was reported. Notably, its structure features an aspartic acid residue in the carboxyl-terminal region, adjacent to the Lan and MeLan rings, making it the first lanthipeptide with this structure (Fu et al. 2023).

### LanM enzymes

The biosynthetic enzyme LanM is responsible for producing class II lanthipeptides. This protein contains two distinct domains within its structure. The N-terminal side catalyzes the dehydration reaction through a mechanism that utilizes adenosine triphosphate (ATP). Meanwhile, the C-terminal side is responsible for the cyclization reaction (Zhang et al. 2012; Yu et al. 2013; van der Donk and Nair 2014). The activity of this enzyme has been determined *in vitro* (Ma et al. 2014; Shimafuji et al. 2015) in *E. coli, Streptomyces* strains (Widdick et al. 2003; Nagao et al. 2005; Boakes et al. 2009; Caetano et al. 2014), and even in mammalian cells (Eslami et al. 2024). The BGC associated with this lanthipeptide class are widely distributed in Gram-positive strains, actinobacteria, and cyanobacteria genomes (Walker et al. 2020). The LanM enzymes in cyanobacteria strains have special features because they can modify various precursor peptides encoded on the same genome with variations in their core sequences. The first promiscuous enzyme was discovered in *Prochlorococcus* MIT9313 (Li et al. 2010) and subsequently described in other genera, such as *Synechococcus* sp. and *Mycrocystis* sp. However, genome mining studies have estimated homologous BGCs in many bacterial groups, including α and γ-proteobacteria (Arias-Orozco et al. 2021). Specifically, a study conducted in *Synechococcus* MITS950 determined that this strain harbors a BGC within seventy-nine precursor peptides and only one LanM enzyme, SyncM (Arias-Orozco et al. 2021). This enzyme represents the biosynthetic enzyme for lanthipeptide production with the most flexible substrate reported. To date, fifteen peptides have been successfully produced with various ring topologies and directions of lanthionine ring formation. However, like class I lanthipeptides, the precursor peptides share a high similarity in the LP sequences, and a limitation of some amino acids that are found in side-to-ring-forming amino acids has been reported. Although this latter limitation in SyncM is lower than that in the enzymes ProcM and SyncWM from *Prochlorococcus* sp. MIT9313 and *Synechococcus* sp. UW179A (Arias-Orozco et al. 2021). A feature of this homologous protein is the “CCG” motif in the cyclization domain, which can generate low specificity for substrates due to its ability to chelate a greater amount of zinc ions (Zhang et al. 2012, 2022; Repka et al. 2017). Kinetic studies on the ProcM enzyme have demonstrated considerable flexibility in tolerating variations in the sequences of precursor peptides, specifically regarding the ring-forming amino acids and those surrounding the rings. However, the reaction rate decreases as the rings are formed (Mukherjee and van der Donk 2014; Thibodeaux et al. 2014, 2015; Yu et al. 2015). A study comparing ProcM with MalM, a homologous LanM enzyme found in *Microcystis aeruginosa* NIES-88, revealed that while ProcM can produce chimeric peptides from MalA (the precursor peptides of MalM), MalM is unable to make modifications to the precursors of ProcM. In contrast, ProcM can modify MalA to generate structural variants (Zhang et al. 2022). These findings suggest that, despite being homologous proteins, LanM enzymes may exhibit selectivity for their specific precursor peptides.

**Fig. 3:**
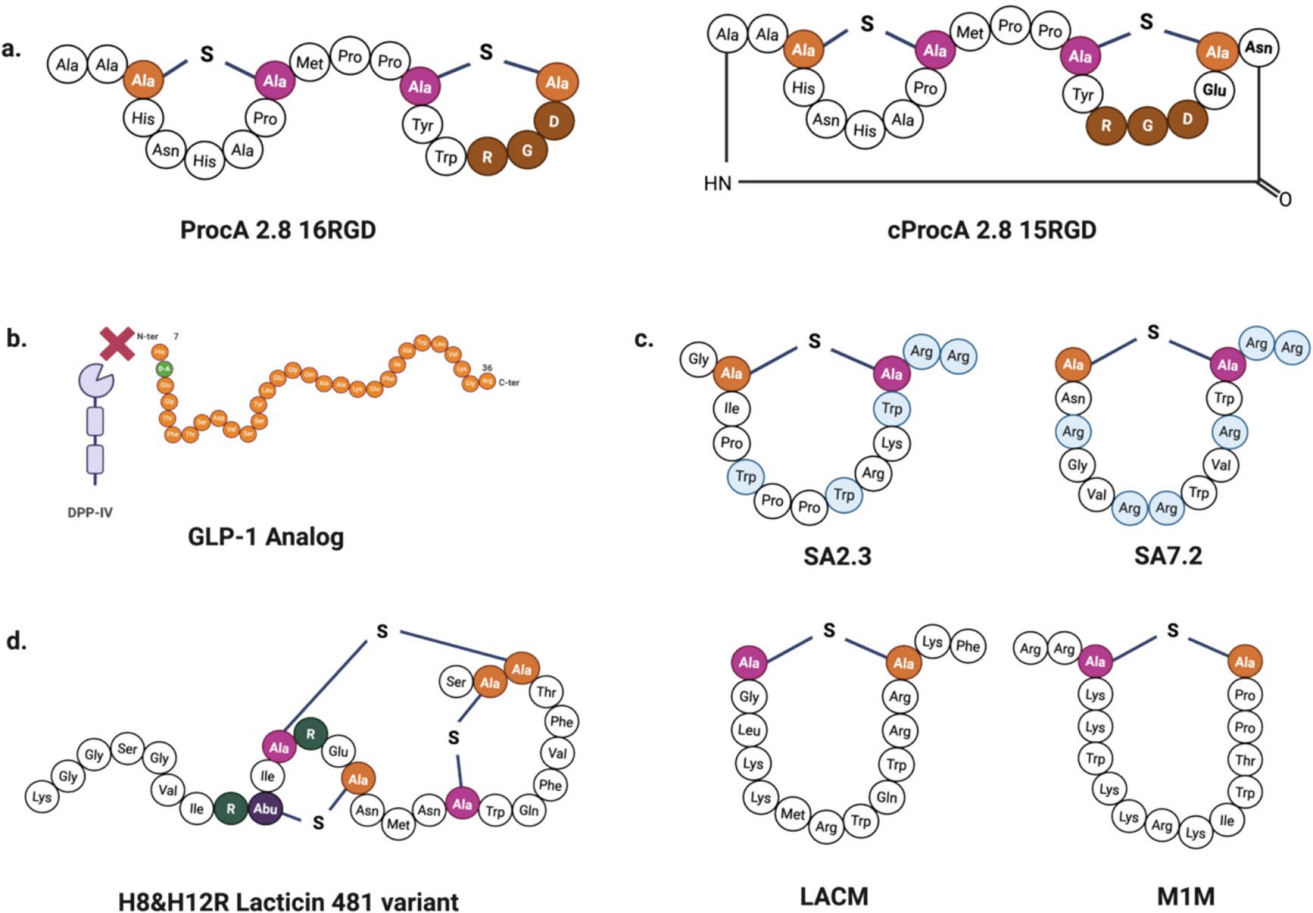
Structures of peptides produced by promiscuous LanM enzymes. **a** ProcA 2.8 16RGD and cProcA 2.8 15RGD are derivative peptides of prochlorosin 2.8, featuring the integrin binding epitope RGD (highlighted in brown) (Hegemann et al. 2019; Le et al. 2024). The latter is a cyclic peptide. **b** The GLP-1 analog produced by ProcM is resistant to proteolysis by dipeptidyl peptidase IV (DPP-IV) (Larsen et al. 2024). **c** The H8&H12R lacticin 481 variant contains ornithine at the 8 and 12 positions (illustrated in green) (Xu et al. 2024). **d** Bioactive cyclic peptides modified by the SyncM enzyme have mutated amino acids in the SyncA precursors, which are highlighted in blue (Arias-Orozco et al. 2023)

Although cyanobacterial lanthipeptides do not exhibit antimicrobial activity, the biosynthetic enzyme ProcM can be utilized to produce diverse lanthipeptide structures with diverse purposes (Table 1). These include libraries for detecting bioactive peptides and, more recently, using lanthipeptides as backbones for peptide display essays to identify ligands. In this context, a 2019 study reported that the addition of the RGD integrin-binding epitope, useful for imaging tumors, to the prochlorosin precursor ProcA2.8 was tolerated by the ProcM enzyme, resulting in the generation of 15RGD and 16RGD peptides (Fig. 3a). These modified peptides showed a higher affinity for the αvβ3 integrin (*Ki* = 1.6 ± 0.3 nM) compared to the linear peptide (*Ki* = 18 ± 3 nM), Moreover, they exhibited resistance to proteolysis to trypsin, with values comparable to other peptides based on lacticin 481, which bind integrins with higher affinity than cilengitide, an established integrin-binding peptide (Hetrick et al. 2018; Hegemann et al. 2019). Later, both peptides were cyclized using an asparaginyl endopeptidase (OaAEP1), an enzyme involved in the macrocyclization of cyclotides. This process produced the aminopeptidase-resistant cyclized peptide c15RGD, which displayed a fourfold higher affinity to the αvβ3 integrin (*Ki* = 7.7 ± 1.3 nM) compared to the non-cyclized peptide (*Ki* = 34.4 ± 6.9 nM) (Le et al. 2024) (Fig. 3a). This approach to peptide display was previously reported, utilizing Lacticin 481 synthetase to produce yeast display libraries. This strategy involved the production of the peptide LctA, which was attached to Aga2 (a subunit of the yeast surface protein agglutinin) within *E. coli*, followed by its subsequent modification in the endoplasmic reticulum. The successful presentation of the lanthipeptide on the yeast surface mirrored previous results and was likewise applied to the HalA2 lanthipeptide, a component of haloduracin (Hetrick et al. 2018).

Urban et al. (2017) performed a study on the presentation of epitopes in bacteria and phages. This study employed the ELISA assay to identify lanthipeptide-like cyclic ligands that bind to the urokinase plasminogen activator (uPA) and streptavidin through phage display. These peptides were produced in *E. coli* and modified by ProcM, and the potential peptide was joined to the C-terminal of the gene-3 minor coat protein (pIII) to form peptide ligands. The results highlighted the flexibility of ProcM in modifying substrates within the N-terminal LP attached to the His-tag and a large fusion protein, such as the maltose-binding protein (MBP). In this regard, the ProcM enzyme is also tolerant to hormone peptide sequences, making it suitable for use in the biomedical field. In 2024, a study reported the modification of glucagon-like peptides (GLP) in *E. coli* by.

ProcM (Larsen et al. 2024). In this process, a specific protein facilitates the dehydration reaction of the GLP-1 peptide. Following this step, a reduction reaction is catalyzed by the dehydrogenase NpnJ, sourced from *Nostoc punctiforme* PCC 73102, resulting in the incorporation of D-Ala8 (D-amino acid) as a modification to the peptide. This modified peptide is resistant to degradation by dipeptidyl peptidase IV (DPP-IV), an enzyme that usually breaks down GLP-1 (Fig. 3b). It is proposed that these modified peptides could be used in therapy for patients with type 2 diabetes (Larsen et al. 2024). In addition to the ProcM enzyme, the SyncM synthetase has been utilized to produce bioactive peptides used by the SyncA, specifically as backbones to produce cyclic antimicrobial peptides (cAP). These peptides are characterized by the presence of positively charged amino acids in their structures. On this basis, the antibacterial peptides SA2.3 and SA7.2 (Fig. 3c), derived from SyncA2 and SyncA7, respectively, were found to be active against the Gram-positive bacterium *L. lactis* N2900. This activity was observed when arginine and tryptophan amino acids were incorporated into their sequences (Arias-Orozco et al. 2023). In the same study, it was found that the SyncM synthetase can modify various non-lanthipeptide substrates. Two peptides derived from lactoferrin, designated as LACM (lactoferricin-derived peptide) and LACPINM (lactoferrampin-derived peptide), were modified by SyncM to generate one-ring cyclized peptides, which exhibited a slight activity against *L. lactis*. Additionally, a peptide derived from murepavadin — a synthetic cyclic Protegrin-1 peptidomimetic antibiotic — known as M1M, was produced and demonstrated activity against *L. lactis* (Arias-Orozco et al. 2023) (Fig. 3c). Additionally, this enzyme was utilized to modify lacticin 481, which contains ornithine, by employing a chimeric LP that combines the C-terminal LP of ProcA3.3 with the LP recognized by OspR. OspR is a peptide arginase that converts arginine to ornithine. The modification process was successful using OspR and later with SyncM at positions 8 and 12 of the lacticin 481 variants (Fig. 3d). These modified peptides demonstrated activity against *B. subtilis* (Xu et al. 2024), underscoring SyncM's remarkable tolerance for non-amino acid substrates, even when the wild-type LP is replaced with the prochlorosin LP. The use of disulfide-bond-based macrocyclic antimicrobial peptides as substrates is a characteristic observed in other lanthipeptide synthetases, such as LanB and LanC. However, additional advantages of LanM enzymes have been reported. SyncM can generate peptides with large rings, accommodating up to fifteen ring-forming amino acids. So far, this enzyme is the most notable synthetase capable of catalyzing the formation of large rings in a lanthipeptide substrate. Furthermore, a fourteen-amino-acid ring was successfully installed on a non-lanthipeptide precursor through SyncM catalysis, with even a C-terminal serine being accepted as a ring-forming amino acid. The presence of positively charged amino acids and prolines was also tolerated. These findings highlight the potential of this enzyme for producing peptide mimics and analogues of cyclic antibiotics (Arias-Orozco et al. 2023).

On the other hand, the mersacidin synthetase MrsM can form the smallest MeLan ring reported to date. In this case, a simple CP of just five amino acids was sufficient to create a two-component ring, retaining only the LP (Viel and Kuipers 2022). Both studies highlight the enzymatic advantages of LanM over other lanthipeptide enzymes.

Researchers have reported the use of other synthetases, such as LanM, to produce bioactive peptides. Zhao et al. (2021) highlighted the versatility of CinM, the biosynthetic enzyme responsible for cinnamycin, by demonstrating its ability to produce an analogous lipidated peptide, known as thanatin (Lantha), which includes the installation of a MeLan ring. This peptide exhibits activity against multiple pathogens, including MRSA, VRE, *A. baumannii*, and *Shigella flexneri*, shedding light on the potential for additional modifications to lanthipeptides. Similarly, the biosynthetic machinery of mutacin II from *Streptococcus mutans* T8 was utilized to create a new homologous antimicrobial peptide by directly inserting the precursor gene into the genome of *S. mutans*. The resulting peptide, Nukacin Spp.2, was successfully modified and demonstrated activity against *Listeria monocytogenes* (Biswas 2023).

### LanKC and LanL enzymes

The BCG of class III lanthipeptides has been experimentally identified in several microbial groups, such as Actinobacteria (Wang and van der Donk 2012; Jungmann et al. 2016), Firmicutes (Xue et al. 2022, 2023a), and Myxobacteria (Rukthanapitak et al. 2024). In contrast, class IV lanthipeptides have only been found to date in the genomes of Actinobacteria and Firmicutes (Walker et al. 2020), although most of the characterization work has been concentrated on Actinobacteria (Goto et al. 2010; Iftime et al. 2015). The enzymes LanKC and LanL modify precursor peptides in classes III and IV, respectively. Both proteins have three domains: a lyase domain on the N-terminus, a kinase domain in the central region, and a cyclase domain on the C-terminus. The main difference between them lies in the cyclase domain, which lacks the catalytic amino acids responsible for zinc chelation in LanKC (Hegemann and Süssmuth 2020). The recognition by the biosynthetic enzymes is attributed to a specific motif in the LP, identified as (θxx)θxxθxxθ (where θ can be L, I, V, M, or T), along with an α-helical structure present in both classes (Hegemann and van der Donk 2018; Wiebach et al. 2020). A special feature of class III is the presence of labionin (Lab) structures, which are the primary modification introduced by LanKC (Hegemann and Süssmuth 2020). Although class III and IV lanthipeptides have been widely studied, our understanding of biosynthetic enzymes and their promiscuity remains limited. In this section, we summarize several reports from the last five years.

The actinobacterial lanthipeptides of class III exhibit limited biological activity as antimicrobials; instead, these peptides have been characterized by their antiallodynic and antiviral effects (Iorio et al. 2014; Prochnow et al. 2020; Blockus et al. 2020). In this context, the lanthipeptide NAI-112 is a peptide that has been widely described due to its application as an antinociceptive for pain relief in a murine model (Iorio et al. 2014). The glycosylation of tryptophan (Trp) in NAI-112 is a unique modification installed by AplG glycosyltransferase. This peptide is produced by *Actinoplanes* sp. and harbors seven genes. The AplKC enzyme is responsible for generating modifications in the AplA peptide, and its activity has been successfully reconstituted in the model microorganism *E. coli* (Sheng et al. 2020). The flexibility of this enzyme was examined through mutations involved in the formation of labionin rings. The study highlighted that these rings could form independently, despite the possibility of creating chimeric peptides. Although there are proposals to use the glycosyltransferase AplG to modify other peptides, this enzyme exhibits limited flexibility because it requires the MeLab rings to close the tryptophan (Trp) side chain in order to install the glycosyl group (Sheng et al. 2020).

On the other hand, the activity of LanL enzyme has been successfully active in *E. coli* (Goto et al. 2010) and *Streptomyces* strains (Iftime et al. 2015), as well as through in vitro assays (Hegemann and van der Donk 2018). Experimental results indicate that the activity of the cyclase domain is independent of the dehydration domain, which includes the kinase and lyase motifs (Hegemann and Süssmuth 2021). A study conducted in 2018 established the in vitro production of venezuelin-like lanthipeptides from the GCF147 family of lanthipeptides. This research demonstrated the versatility of LanL enzymes in modifying the precursor peptide of globisporin SgbA from *S. globisporus* subsp. *globisporus* NRRL B229, highlighting the similarity between the sequences of LP (Hegemann and van der Donk 2018).

### LanK, LanY and LanX enzymes

These lanthipeptides are being studied for their antibacterial activity against MDR bacteria. Initially, three members of this class were described: a lexapeptide (Xu et al. 2020), pristinin A3 (Kloosterman et al. 2020) and cacaodin (Ortiz-López et al. 2020). To date, these class V lanthipeptides have only been found in the actinobacteria group, similar to tryantimycins in *Streptomyces* TN58 (Ding et al. 2024), which encodes three precursor peptides in the same BGC. In general, the BGCs of these peptides are large, featuring the main biosynthetic enzymes: LanK (which has kinase activity), LanY (which has lyase activity responsible for dehydration reactions), and a putative cyclase, LanC, responsible for cyclization. However, the presence of LanC is not always necessary for processing all peptides (Pei et al. 2022; Ding et al. 2024). Due to their recent discovery, research on these promiscuous enzymes is currently limited to a few reports. The enzymes are typically produced in heterologous hosts such as *E. coli* and *Streptomyces* strains (Xu et al. 2020; Román-Hurtado et al. 2021; Cheng et al. 2024). Furthermore, the dehydration enzymes are tolerant to purification tags, such as His-Tag, which have been used in the production of cacaodin and SmoA peptides (Deisinger et al. 2023; Wang et al. 2025). Tags for solubilization, like SUMO, have also been successfully attached to precursor peptides, like the lexapeptide and cacaodin produced in *E. coli* (Xu et al. 2020; Xue et al. 2023b).

The cacaodin biosynthetic enzymes represent the main model for understanding the enzymatic challenges associated with this lanthipeptide class. The LanK and LanY enzymes correspond to the dehydration domain of the LanL enzyme, acting as aminoglycoside phosphotransferase and phosphoSer/phospoThr lyases, respectively (Ortiz-López et al. 2020; Xu et al. 2020; Román-Hurtado et al. 2021). The CaoK (kinase) and CaoY (lyase) enzymes form an *in vitro* heterodimer complex to dehydrate the precursor peptide of cacaoidin (CaoA). This process is akin to the NisBCT complex involved in nisin production, where CaoK binds to the PL and CaoY binds independently to the precursor peptide (Liang et al. 2022; Xue et al. 2023b). Although the dehydration complex can dehydrate shorter core peptides, it has not been successful in demonstrating promiscuous activity with other non-native substrates (Liang et al. 2022).

The cyclization reaction is a crucial step in classifying class V lanthipeptides. Not all peptides undergo cyclization by a specific cyclase enzyme; some can cyclize spontaneously or through an undefined mechanism. This has been observed in the production of lanthipeptides in strains of *E. coli* and *Streptomyces*. The biosynthetic pathways include the synthesis of pristinin A3, lexapeptide, cacaoidin, and tryantimycins, all of which are classified as part of the Va subgroup of lanthipeptides. In contrast, a Vb subgroup has also been identified, in which the cyclization reaction is catalyzed by a specialized enzyme known as LanC. Pei et al. (2022) reported the first cyclase enzyme capable of producing the SmoA lanthipeptide. The structure of SmoA features energetically unfavorable overlapping rings, which differ from those found in class Va lanthipeptides. In the same study, SmoA was produced without the SmoC cyclase, resulting in a structure with different ring patterns and stereochemistry compared to the cyclized peptide produced with the SmoC enzyme. While the biological activity of SmoA has not yet been determined, ongoing research into the relationship between ring structures and bioactivity exists. Additionally, the presence of the AvyMeCys group in Va lanthipeptides, which is absent in the Vb group, may be essential for antibacterial activity, regardless of the stereochemistry of the rings. Furthermore, the versatility of the SpcC enzyme—an orthologous enzyme to SmoC found in *Streptomyces pathocidini* NRRL B-24287—was demonstrated when the SmoA peptide was produced with the same structure as that modified by SmoC. This finding suggests that cyclase enzymes are adaptable and can produce peptides with overlapping rings, even when using the same LP as their homologous sequences (Pei et al. 2022). Class VI lanthipeptides, like their class V counterparts, have only been identified within the *Streptococci* phylum (He et al. 2023). Because they were only recently discovered, there have not yet been any studies examining the promiscuity of LanKCb enzymes, which are responsible for introducing post-translational modifications. However, it is noteworthy that LanKCb has had successful activity in vitro.

### Proteases and secondary tailoring enzymes

The versatility of biosynthetic enzymes goes beyond the primary proteins to include secondary tailoring enzymes and proteases (see Table 2). Proteases are essential for removing the LP, which results in the formation of the mature peptide. Meanwhile, secondary tailoring enzymes are responsible for adding chemical modifications to specific amino acids on the precursor peptide, and many of these functions can occur independently of the LP. In this section, we will explore how these enzymes can be utilized to produce additional modifications and facilitate the proteolysis of modified peptides.

**Table 2:** Requirements of promiscuous proteases and some secondary tailoring enzymes involved in the production of bioactive peptides. The tailoring enzymes are considered leader peptide-independent

Modifying enzyme	Activity	Requirements and characteristics	References
NisP	Proteolysis of the leader peptide	An enzyme with in vitro activity showing high flexibility in its recognition sites. It can recognize sequences such as Factor Xa and thrombin with seemingly no restrictions on the core peptide. This makes it helpful in producing different classes of lanthipeptides	(Montalbán-López et al. 2018; Arias-Orozco et al. 2021)
AprE		Recognition of the double-glycine motif (GG) in the leader peptide enables a semi-pure enzyme to participate in the proteolysis reaction	(Corvey et al. 2003; Fu et al. 2023, 2024)
LahT150		The ability to cleave peptides with a double-glycine motif includes the Nif11 family. However, some restrictions on Lysine (Lys) and Aspartic acid (Asp) at the −4 and −7 positions of the leader peptide have been reported	(Bobeica et al. 2019)
AlpP		There is flexibility in cleaving the leader peptides of actinobacterial class III lanthipeptides	(Chen et al. 2019)
CaoSc	Methylations in the core peptide	The only requirement for one specific enzyme is the presence of a dehydrated amino acid (Dhbx) in the second position of the core peptide	(Liang et al. 2024)
MicD	Oxidation of the C-terminal cysteine	Flavin-dependent cysteine decarboxylase with in vitro activity	(Xia et al. 2023)
NpnJA	Reduction of dehydrated amino acids	NADPH-dependent dehydrogenase has in vitro activity to reduce Dha to D-Ala	(Yang and van der Donk 2015)
RodJA		NADPH-dependent dehydrogenase with the capacity to modify both Dha and Dhb residues	(Fu et al. 2024)

As previously mentioned, the NisP protease is responsible for cleaving the NIS. This enzyme is classified as a subtilisin-like serine protease, as demonstrated in several studies. It catalyzes the proteolysis of the LP in NIS, forming the mature peptide (Cheigh and Pyun 2005; Lubelski et al. 2008). The reaction occurs when NisP recognizes the motif GASPR|IT between the leader and core peptides (Lagedroste et al. 2017). Several studies have indicated that NisP can also recognize other peptidase motifs, such as those of Factor Xa and thrombin (Montalbán-López et al. 2018). These findings suggest that NisP is a promiscuous enzyme, capable of catalyzing proteolysis even when an arginine residue is in the −1 position. Notably, this proteolysis can occur without the Lan or MeLan ring in the LanA peptide (Montalbán-López et al. 2018). To date, no studies have reported the use of these sites for the proteolysis of lanthipeptides. However, NisP's low specificity towards the core region has enabled the insertion of its cognate site into several precursor peptides, such as synechococcins from *Synechococcus*, without affecting post-translational modifications (Arias-Orozco et al. 2021).

The ABC transport family is responsible for the transport process in class II lanthipeptides. These proteins contain a C39 cysteine protease domain at the N-terminus, which often facilitates the proteolysis of the LP after the"GG"motif (GG/GA/GS) in the LP (Barbosa et al. 2015; Eslami and van der Donk 2024). While these proteins can be separated, it is noteworthy that transporter proteins can export various molecules, including non-lanthipeptides. For example, in a study involving the LicT and LicP enzymes—responsible for the export and proteolysis of lichenicidin, respectively—researchers synthesized peptides insulin A and amylin, which are known to regulate glucose levels. These peptides were fused to the LicA2 leader, a component of lichenicidin. The resulting fusion proteins were successfully exported and digested through *E. coli* membranes by LicTP proteins without alteration. These findings were also observed in the transport of epidermin, a class I lanthipeptide (Barbosa et al. 2023). This supports the utility of this system for exporting biomedical interest peptides produced in *E. coli.* The proteolysis of the LP is often catalyzed by nonspecific extracellular proteases in Firmicutes strains, such as mersacidin, a lanthipeptide known for its potent antibacterial activity. In the case of mersacidin, the proteolysis of the LP occurs in two steps, facilitated by the bifunctional MrsT protein and the protease AprE, resulting in the formation of active mersacidin (Viel et al. 2021a, b). AprE is a subtilisin-like protease produced by several *Bacillus* strains and was first identified in *B. subtilis* ATCC 6633 as one of the key proteases involved in the biosynthesis of subtilin (Corvey et al. 2003). The similarity between AprE and other proteases from *Bacillus* has enabled the use of AprE for the heterologous production of various lanthipeptides in a one-step process following the GG motif. In the case of the two-component lanthipeptide rodecin, derived from *B. subtilis* EH5, a semi-purified version of AprE from *B. subtilis* 168 was utilized for the in vitro cleavage of the leaders RodA1 and Rod2, even without the proteolysis of RodT (Fu et al. 2024). The same protein was utilized to produce balucin, a class I lanthipeptide, which provides an accessible method for cleaving the LP from peptides derived not only from *Bacillus* species but also from others, such as *Clostridium estertheticum*. In this process, AprE from *B. amyloliquefaciens* was cleaved to yield estercin A, a lantibiotic effective against MRSA (Wang et al. 2025).

One commonly used protease in the production of lanthipeptides is LahT150. This protease domain is derived from the bifunctional transporter/protease protein, LahT, found in the *Lachnospiraceae bacterium* C6A11. LahT150 can cleave its substrates as well as prochlorosins, haloduracin, lacticin 481, and peptides from the Nif11 family that contain the GG motif. However, it restricts explicitly cleavage positions to −4 and −7 within the 13 C-terminal sequence of the LP (Bobeica et al. 2019). The use of LahT150 has been reported in various applications, including the cleavage of cyclic ligands to αvβ3 integrin utilizing ProcA2.8 as a backbone (Le et al. 2024) and for PedA15.2, a new class I lanthipeptide derived from *Pedobacter lusitanus* NL19 (Bothwell et al. 2021). In 2022, LahT150 was utilized in the automated production of non-characterized lanthipeptides on a platform called FAST-RiPPs, leading to the discovery of new lantibiotics (Ayikpoe et al. 2022).

The proteolysis of the LP in class III lanthipeptides is carried out by two different enzymes: an endopeptidase, which cleaves the first amino acid on the amino-terminal side, and an aminopeptidase, which is responsible for removing the remainder of the LP (Hegemann and Süssmuth 2020). In the production of NAI-112, the metalloenzyme AlpP functions as a bifunctional enzyme, performing both proteolytic activities.

A study by Chen et al. (2019) demonstrates the flexibility of AlpP, as it can cleave the LP in other precursors, such as erythrapeptin, SapB, and flavipeptin. This suggests that it has the potential to be a useful enzyme for generating another class III lanthipeptide. Similar proteases can be found in other organisms, such as *Streptomyces lividans* TK24, which can partially remove the LP. Zinc-dependent proteases are also present in other bacteria, including *E. coli*. This feature allows for the cleavage of the LP in lanthipeptides produced in this host, creating an opportunity to express biosynthetic gene clusters (BGCs) without requiring a specific protease. However, a downside of this approach is the potential for nonspecific cleavage in the modified peptide. This was demonstrated in the production of boletupeptin, a new lanthipeptide derived from the myxobacterium *Melittangium boletus*, where a nonspecific protease catalyzes cuts at two different sites. This issue may also arise with class IV lanthipeptides, as Ren et al. (2020) reported.

Secondary tailoring modifications in lanthipeptides involve the introduction of specific chemical groups onto precursor peptides, including methylations, oxidations, reductions, lipidations, and glycosylations. In RiPPs, these modifications can enhance the pharmaceutical properties of peptides, including their solubility, bioactivity, and stability (Funk and van der Donk 2017). In this context, the characteristics of modified lanthipeptides may be advantageous, depending on the specific modifications made, although in some cases, the bioactivity could be compromised. For example, Andalusicin A, a methylated lanthipeptide, exhibits antimicrobial activity only when a di-methylation occurs at the N-terminal side (Grigoreva et al. 2021). Microbisporicin, another potent antibacterial lanthipeptide, contains a halogenated tryptophan residue (ClTrp) and a C-terminal S-[(Z)−2-aminovinyl]-D-cysteine (AviCys) (Castiglione et al. 2008; Ortega et al. 2017). It has been observed that halogenation is crucial for antibacterial activity; even a substitution of chlorine with bromine can enhance bioactivity (Cruz et al. 2015). A molecular dynamics simulation of cinnamycin interacting with phosphatidylethanolamine (PE) lipids demonstrates that the hydroxylation of Asp15 interacts with this structure, which is an important target for its antiviral and antimicrobial activity, as well as its applications as a molecular marker (Vestergaard et al. 2019). Specific enzymes are encoded near the primary modification enzymes and the precursor peptide. Several representative cases demonstrate these proteins'versatility in modifying other lanthipeptides and non-lanthipeptides (Ortega et al. 2014; Larsen et al. 2024).

Methylations are added by specific methyltransferases found in biosynthetic gene clusters (BGCs) from Firmicutes and Actinobacterial origins (Xue et al. 2023a). In Firmicutes, these methylations are carried out by S-adenosylmethionine (SAM)-dependent methyltransferases. These modifications can enhance antibacterial activity against certain bacteria, such as *Bacillus* strains and *S. aureus,* when methyl groups are added to the N-terminal side of precursor peptides (Grigoreva et al. 2021; Xue et al. 2023a). Employing these enzymes for the methylation of other substrates has been suggested.

Methyltransferases have specific requirements for precursor peptides. Notably, dehydrated amino acids must be present in the second position, such as 2,3-dihydroxybutyrate (Dhb) or dehydroalanine (Dha). In contrast, there is more flexibility regarding variations in the first position of the CP. This approach has been successfully implemented for paenithopeptins derived from *Paenibacillus thiaminolyticus* NRRL B-4156 (Xue et al. 2023a). Moreover, in the same study, a mutation was introduced in PttA1, one of the precursors of paenithopeptin, which is not naturally methylated. After changing the second position to threonine (Thr), this precursor was successfully methylated (Xue et al. 2023a). Conversely, the methyltransferase CaoSc adds two methylations to the N-terminal side of cacaoidin after LP removal. Recent research indicates that only a Dhb residue in the −2 position on the N-terminal side is needed for the modifications to be installed (Liang et al. 2024). Additionally, this enzyme exhibits in vitro activity with a broad range of substrates, including nisin and cytolysin, a two-component class II lanthipeptide. It successfully installs a methyl group on the N-terminal side of the peptide. In the case of Hulth's Halβ peptide, the introduction of two methyl groups by CaoSc significantly enhanced its antibacterial efficacy against *Lactococcus lactis* subsp. *cremoris*, while also contributing to increased solubility in the surrounding medium (Liang et al. 2024). Although the precise mechanisms underlying this improvement in solubility remain somewhat unclear, the observed increase in antibacterial activity may be attributed to enhanced membrane penetration, which is likely a result of increased lipophilicity of the substrate. Additionally, this modification could help prevent degradation by aminopeptidases, thereby preserving the peptide's functionality (Xue et al. 2023a; Liang et al. 2024).

In class V lanthipeptides, tailored modifications, such as aminovinyl-(methyl)cysteine (Avi(Me)Cys)—play a crucial role in enhancing their activity. This is achieved through the formation of a large lipid II-lanthipeptide complex, which disrupts the cell wall (Sit et al. 2011; Maffioli et al. 2015; Escano et al. 2017; Chu et al. 2022). In contrast, class I lanthipeptides have been shown to possess a chemical group that protects against carboxypeptidases, and the activity of the decarboxylase in this class is not dependent on the LP (Maffioli et al. 2015; Ortega et al. 2017). Another significant modification, such as methylation in cacaodin, can also be essential for altering the solubility and overall activity of the peptides. The Avi(Me)Cys modification is incorporated into several class V lanthipeptides by a specialized flavoprotein, like those found in class III lanthipeptides.

A study conducted by Cheng et al. (2024) reported the establishment of a complete system for class V lanthipeptides, which includes flavoproteins and O-methyltransferases, to produce new peptides from families of homologous sequences derived from strains of *Streptomyces*. The resulting peptide, or sisteride A2, can be modified by enzymes to form lanthipeptides, such as kebanetides, using the same LP. This process also leads to the generation of new methylated variants, showcasing the versatility of the flavoprotein system (Cheng et al. 2024).

Another example is microvionin, a member of the lipolantine group with activity against MRSA. This peptide features the S-[(Z)2-aminovinyl]-D-cysteine (AviCys) modification, which is carried out by the MicD enzyme, a flavin-dependent cysteine decarboxylase (Wiebach et al. 2018; Grant-Mackie et al. 2021). MicD demonstrates *in vitro* activity and has been shown to oxidize the C-terminal cysteine of the precursor MicA (its cognate substrate) when linked to GFP. It has also facilitated the generation of fluorescence-labeled proteins after incubating MicA-GFP, MicD, and rhodamine B (Rhb), highlighting the potential of (thio)aldehydes for bioconjugation.

Furthermore, oxidized MicA, along with shorter MicA peptides and two synthetic peptides (Pep-1 and Pep-2), spontaneously generated a head-to-tail cyclic peptide *in situ* after modification by MicD (Xia et al. 2023).

On the other hand, the reduction reactions are commonly reported in lanthipeptide biosynthesis. These modifications involve the hydrogenation of Dha and Dhb to produce D-amino acids. The NpnJA enzyme is a NADPH-dependent dehydrogenase and was the first dehydrogenase with *in vitro* activity reported. This protein from *N. punctiforme* PCC 73102 is tolerant to modification on its cognate precursor, NpnA3, and selectively reduces Dha to D-Ala. These enzymes are leader-independent when modifying another substrate.

LtnA1 and LtnA2, components of lacticin 3147, were successfully reduced by one and two-fold in *E. coli* production alongside NpnJA. Additionally, a non-cycled variant of lacticin 481 served as a substrate for the dehydrogenase. Two randomized peptides, PA1 and PA2, underwent dehydration by ProcM, which contains D-Ala after reduction by NpnJA (Yang and van der Donk 2015). The higher tolerance exhibited by NpnJA has proven beneficial in producing structures such as glucagon-like peptides, as previously discussed (Larsen et al. 2024), along with other peptides. One example is dermorphin, a heptapeptide that acts as an agonist of μ-opioid receptors. This process involves the BsjM enzyme, a biosynthetic enzyme derived from bicereucin, and NpnJA, which together form a demorphin variant with one reduction (Huo and van der Donk 2016). The principal limiting factor in utilizing NpnJA is its selectivity for Dha residues.

Rodencin, a two-component class II lanthipeptide, is modified by a NADPH-dependent dehydrogenase known as RodJA. This enzyme can reduce both Dha and Dhb, a capability that has not been previously documented. In a promiscuity test, RodJA successfully reduced the Ltnb variant of lacticin 3147, which has a Ser12Thr substitution, on two occasions. The ability to modify amino acids in lanthipeptides can be applied to other substrates to enhance antimicrobial activity, potentially as seen in rodencin through its interaction with lipid II, although this mechanism is still not fully understood (Fu et al. 2024). Additionally, these modifications may improve resistance to proteases (Yang and van der Donk 2015).

### Final comments and prospects

The biosynthesis of lanthipeptides in producer strains still represents substantial challenges for researchers. The often low or non-existent production levels and the complexities of purifying and identifying these peptides demand considerable effort and resources. In this context, the expression of these peptides in heterologous hosts, such as *E. coli*, represents a promising strategy for enhancing production. However, the existence of bottlenecks such as the formation of inclusion bodies and the failure to replicate essential post-translational modifications in alternative strains, which can lead to insoluble proteins, underscores the urgent need for innovative platforms to generate new bioactive peptides. Overcoming these hurdles is crucial for advancing the field and unlocking the full potential of lanthipeptides, emphasizing the utility of other platforms in generating new bioactive peptides.

The promiscuity of biosynthetic enzymes represents a powerful and innovative approach for expressing entire BGCs within heterologous hosts. As highlighted in this review, the increasing repertoire of synthetases adept at modifying precursor peptides paves the way for the rapid generation of diverse peptide libraries, leading to the discovery of bioactive molecules in remarkably short time frames. In this context, understanding the enzymatic kinetics and the functionality of LP is crucial for designing new peptides. LP plays a controlled role in enhancing the efficiency of peptide modifications. Techniques such as isothermal titration calorimetry (ITC), co-purification, and surface plasmon resonance (SPR) demonstrate the high affinity of this peptide segment for processing CP (Mavaro et al. 2011; Uggowitzer et al. 2021; Knospe et al. 2023). The creation of enzymes linked to LP, referred to as the ConFusion enzyme, supports the utility of LP in improving yield and efficiency. Although ProcM has shown broad substrate promiscuity, its reaction rate is slow compared to other synthetases, such as HalM2, which is the haloduracin synthetase (Thibodeaux et al. 2016). While HalM2 exhibits higher substrate specificity, even with HalA1, the component of haloduracin, one may question whether enzyme promiscuity is advantageous. Ecologically, the production of multiple lanthipeptides, such as SycnA's, plays a competitive role in their interactions with similar strains, suggesting that catalysis with a simpler enzyme may be an effective evolutionary strategy (Arias-Orozco et al. 2025). Structurally, this also has new implications. Recently, a crystal structure of MadC (the cyclase associated with maddinglicin) revealed a b-sheet domain, which likely contributes to specific substrate recognition due to its absence in NisC, one of the most promiscuous cyclase enzymes (Knospe et al. 2023). From a biotechnological perspective, these enzymes are increasingly important, as they enable the generation of various biologically active peptides rather than just a single lanthipeptide. Furthermore, genomic and phylogenetic analyses bolster the potential to refine peptide structures, often significantly enhancing their antibacterial properties. For instance, in a phylogeny-based genome mining study of the two-component lanthipeptide roseocin, a variant demonstrated a markedly lower MIC against methicillin-sensitive *S. aureus* (Chaudhary et al. 2023).

Today, integrating robotic systems and cutting-edge methodologies has unlocked new avenues to harness enzymatic promiscuity fully. A 2022 study showcased a robotic work cell that successfully produced over 1300 variants of the Halα peptide through a semi-automated process leveraging the versatile HalM1 enzyme and the LicP protease (Guo et al. 2022). Notably, variant peptides with modifications in single positions are typically well accommodated by the predominant enzymatic proteins. Moreover, incorporating innovative methodologies utilizing supplemented media enriched with non-canonical amino acids—specifically azidohomoalanine, norleucine, and ethionine—has successfully produced a NIS variant where ethionine replaces methionine, achieving complete modification by NisBCTP. Furthermore, this study employed a cutting-edge *in vitro* click chemistry approach, yielding a lipidated nisin that effectively targets vancomycin-resistant enterococci (Guo et al. 2023). This strategy holds significant promise for enhancing the functionality of variant peptides. In 2024, a groundbreaking variant of NIS featuring a benzyl group-containing tail, introduced through non-canonical amino acids and refined via click chemistry, demonstrated remarkable stability against proteolytic degradation compared to traditional NIS. While this novel peptide exhibited reduced activity, its enhanced stability marks a pivotal advancement in peptide development (Guo et al. 2024a).

Not only are the main biosynthetic enzymes used to generate new peptides, but the secondary tailoring enzymes also play a crucial role in the variations in activity. Punctual mutations have been crucial in demonstrating the independence of the LP in these proteins. Despite the potential for use, other techniques are being tested to insert functional groups. A similar methodology used to generate an ethionine-NIS variant was equally used to produce a bromotryptophan-labeled nisin with a twofold activity reduction against MRSA concerning wild-type NIS (Guo et al. 2024b).

A new objective in this field is to mimic the production of non-ribosomal peptides, which are known for their stability and high activity against Gram-negative strains. RiPep2, a synthetic version of brevicidine produced through NIS machinery catalysis, demonstrates antibacterial activity against *Xanthomonas campestris*—an atypical effect for lanthipeptides (Zhao et al. 2020a). Additionally, SyncM and PirF, which catalyze lipid modifications, could help create mutant variants of brevicidine that serve as novel antibacterial agents, paving the way for new non-natural peptides (Xu et al. 2025).

Biosynthetic enzymes are fundamental drivers in the production of a diverse array of bioactive peptides, each with significant potential across various applications. Recent mechanistic and kinetic studies have highlighted critical requirements for essential post-translational modifications, underscoring their importance in this field. The rapid discovery of novel families and lanthipeptide structures presents an urgent need for extensive enzymatic research to enhance the characterization of engineered peptide variants. This, in turn, will pave the way for developing groundbreaking and superior peptide products. We can further accelerate this progress by harnessing cutting-edge techniques such as omics approaches and automation. Research in this vital area is poised for continued exploration and innovation in the years ahead.

## Data Availability

No datasets were generated or analysed during the current study.
